# A randomized controlled trial of different serum phosphate ranges in subjects on hemodialysis

**DOI:** 10.1186/s12882-019-1216-2

**Published:** 2019-02-04

**Authors:** Ramya Bhargava, Philip A. Kalra, Mark Hann, Paul Brenchley, Helen Hurst, Alastair J. Hutchison

**Affiliations:** 1 Upstate Medical University, 750, East Adams Street, Syracuse, NY 13210 USA; 2 Salford Royal Hospitals NHS Foundation Trust, Stott Lane, Salford, UK; 3 University of Manchester, Oxford Road, Manchester, M13 9WL UK; 4 Manchester Institute of Nephrology and Transplantation, Oxford Road, Manchester, M13 9WL UK; 5 Manchester Academic Health Science Center, Manchester, UK; 6 Dorset County Hospital, Dorchester, UK

**Keywords:** Hyperphosphatemia, Oral phosphate binders, Clinical trial, Dialysis, Mortality

## Abstract

**Background:**

Hyperphosphataemia in dialysis subjects is associated with increased mortality. However cause and effect has not been proven, and the ideal phosphate target range is unknown despite KDOQI’s call for studies over 12 years ago. The design and conduct of a randomized controlled trial is challenging because maintaining two groups within differing target ranges of serum phosphate has not been achieved over a long follow-up of 1 year, in a trial setting, before. The SPIRiT study examines the subject acceptance, recruitment and retention rates for such a study in which subjects were randomised to two distinct serum phosphate concentrations, then titrated and maintained over 12 months.

**Methods:**

A two center trial of 104 hemodialysis subjects randomized to lower range LRG 0.8–1.4 mmol/L or 2.5–4.3 mg/dL) and higher range (HRG 1.8–2.4 mmol/L or 5.6–7.4 mg/dL) serum phosphate groups. Two months’ titration and ten months’ maintenance phase. Interventions were non-calcium phosphate binders, self-help questionnaires, with blood tests at specified time intervals.

**Results:**

Thirteen percent of the eligible dialysis population were successfully recruited. A mean separation by serum phosphate of 1.1 mg/dL was achieved and maintained between the groups over 10 months. Drop-out rate was 27% with mortality 10%. Nine subjects in the HRG (17.6%) and two subjects in the LRG (3.8%) died during the study, however the study was not powered to detect significant differences in outcomes.

**Conclusion:**

Randomizing dialysis subjects to separate treatment targets for serum phosphate can achieve a clinically significant sustained separation over 12 months. A large scale longer term study is required to examine outcomes including mortality.

**Trial registration:**

The trial registration number is ISRCTN24741445 – Date of registration 16th January, retrospectively registered.

## Background

Large observational studies have identified hyperphosphatemia as an independent risk factor for cardiovascular disease and mortality in dialysis patients [1–3]. Such studies cannot establish causality, but current guidelines encourage careful control of serum phosphate in the uncertain expectation that it will improve outcomes. Studies of serum phosphate focus on licensing requirements for the binder [4–11] rather than the benefits of lowering serum phosphate per se, and have not addressed the fundamental questions of why, and to what target, serum phosphate should be controlled [12, 13]. Despite significant investment in expensive oral phosphate binders [14–19] (up to $4500 per month per patient) [20], large pill burden (up to 15 pills daily) and significant rates of non-adherence [18], there remains no evidence that lowering serum phosphate definitely improves clinical outcomes.

Randomising subjects to a binder or placebo is unlikely to gain ethical approval for anything other than a short-term study, but we propose that a randomised controlled study of different concentrations of serum phosphate would answer the crucial questions around the value of controlling serum phosphate. However, whether it is possible to achieve and maintain low concentrations of serum phosphate in the range required to enable comparison with a higher concentration, and whether it is possible to maintain the phosphate concentration separation between the groups over an extended time period, is unknown. Furthermore, the consent rate for a trial in which subjects would be randomised to a treatment target above the current guidelines needs to be assessed.

We report the results of a study to examine these factors and to determine whether a large scale, long term randomized controlled outcome trial (RCT) may be possible. The Serum Phosphate Intervention in Renal Replacement Therapy (SPIRiT) trial randomised 104 hemodialysis patients to two groups with different serum phosphate target ranges; a Lower Range Group (LRG) and a Higher Range Group (HRG). Initial screening involved all patients from the two adult renal services of Greater Manchester in England, a conurbation with a total population of 2.8 million people.

## Methods

All trial procedures adhered to the Declaration of Helsinki and the trial protocol was reviewed and approved by the National Regional Ethics Committee East Midlands Derby, REC Ref No. 13/EM/0042.

Details of the SPIRiT trial objectives, design and methods have been published previously [21]. It is an open-label, dual center, randomized controlled trial in which all hemodialysis patients from two large adult renal centers in Manchester (centers 1 and 2) had their electronic medical records screened. All in-center HD patients aged 30 years or above, on regular dialysis for at least 6 months (to ensure no recovery of renal function), meeting UK Renal Association standards for quality of dialysis, with a 3-month mean serum phosphate > = 1.4 mmol/L (4.3 mg/dL) despite binders, serum PTH of < 900 pg/L, and able to consent, were deemed eligible. All eligible subjects were approached for consent. Written Informed consent was obtained from subjects who agreed to enter the study and baseline characteristics recorded. Their current phosphate binder was discontinued and serum phosphate concentration checked after a 3 week washout period. The washout period ended if the phosphate concentration rose to > = 1.7 mmol/L (5.3 mg/dL) but could continue for a maximum of 5 weeks. Those whose serum phosphate concentration did not reach 1.7 mmol/L (5.3 mg/dL) were excluded from randomisation. All blood samples were collected before the commencement of a dialysis session (pre-dialysis samples). All subjects received a single dietetic review and advice regarding control of dietary phosphate intake.

### Randomization

One-hundred and four dialysis patients were randomised in SPIRIT. It was anticipated that 70% of these would be recruited from study center 1 and its satellite centers, with the remaining 30% from study center 2 and its satellite centers. A separate randomisation schedule was generated by the study statistician, using STATA (V12) statistical software – for each of the two study ‘sites’. Only the study statistician had access to these, which were held on a password-protected drive on the central server at the University of Manchester.

To minimise imbalance in the number of patients allocated to either the lower phosphate range group or the upper phosphate range group, block randomisation was employed, using blocks of size 4 and 6 in a random order. Within each block, trial participants were randomly allocated to one or other of the groups with equal probability. As the trial was not blinded (both patients and the research team were not blind to group allocation, given the nature of the intervention), this strategy guarded against the research team being able to predict the next allocation in the sequence.

No formal sample size calculation has been conducted given the exploratory and evaluative nature of the study. The data analysis is largely descriptive and therefore the statistician was also not blinded to the intervention.

## Primary endpoint

The percentage of study participants achieving, and being maintained within, the higher and lower target ranges for serum phosphate, over the duration of the maintenance phase of the study.

## Secondary endpoints

1. Percentage of eligible invited participants willing to be randomised into a study which includes a ‘higher range’ group.
2. Percentage of participants achieving consistent control of serum phosphate in each group over a 10 month maintenance period.
3. Drop-out rate from the study due to adverse events, kidney transplantation, inter-current illness, death. These numbers will inform the power calculation for the larger national study.
4. Pill burden per participant required to control serum phosphate.
5. Incidence of major vascular events, defined as non-fatal myocardial infarction or any cardiac death, any stroke, or any arterial revascularisation excluding dialysis access procedures (expected incidence of around 5% per annum in patients on dialysis).

Randomisation was to the lower range group (LRG with treatment serum phosphate target of 0.8 to 1.4 mmol/L - 2.5-4.3 mg/dL) or higher range group (HRG with treatment target of 1.8 to 2.4 mmol/L - 5.6–7.4 mg/dL). The ‘titration phase’ comprised 2 months following randomization during which the subjects were prescribed variable doses of Lanthanum or Sevelamer (if necessary), in order to achieve study-group targets. Serum phosphate concentration was measured once a week during this phase with dose-adjustment of phosphate binder.

### Study medication

Non-calcium containing phosphate binders were used since calcium containing binders could potentially confound the results because of differences in calcium load between the two groups [22]. Subjects had a choice between Lanthanum and Sevelamer and between tablet and granule preparations. Subjects could change from one formulation to another during the study if they felt unable or unwilling to take a particular formulation. The medication was supplied to them at their dialysis sessions, and advice regarding the change in dosage and change in the timing of the tablets was provided either in person, over the telephone or through the staff on the dialysis unit.

### Maintenance phase

The maintenance phase started after the titration and lasted 10 months. The study conditions closely mirrored standard clinical care. Subjects had a pre-dialysis blood sample once a month for serum phosphate, calcium, albumin and cholesterol concentrations, and PTH measurements were performed every 3 months. The dose of phosphate binder was adjusted to maintain the serum phosphate in the target range of the study group. Each study visit coincided with their normal dialysis session and information regarding serious adverse events (SAE) was collected at the visit or by telephone. Study staff also sought additional information from hospital records and clinical staff regarding all reports of SAEs including myocardial infarction, stroke, death, other causes of hospital admission and fistula thrombosis.

### Statistical analysis

A sample size calculation was not performed for this study given that its primary purpose was to assess issues of design of a larger study to establish whether phosphate concentrations influenced clinical outcomes. Therefore statistical analysis was descriptive or exploratory in nature, presenting appropriate summary statistics only (with 95% confidence intervals where necessary). No inferential conclusions were drawn via reference to test statistics or *p*-values. Analyses were performed using STATA (v13) and Graphpad Prism Version 7.0.

Recruitment rates were calculated for the available hemodialysis population. Continuous demographic, health and blood-related variables were summarised using mean and standard deviation (SD) or median and inter-quartile range (IQR), dependent on their distribution. An exploratory Cox regression analysis was performed to calculate hazard ratios and their 95% confidence intervals after adjusting for age, duration of dialysis, pre-randomization diabetes status and cardiovascular disease.

## Results

### Randomization

Fifty-one subjects were randomized to the HRG and 53 subjects to the LRG. Table 1 shows the baseline demographics and biochemistry. No stratification for known risk factors was employed because of the small numbers randomised, with the result that the LRG were 5 years older but had 0.5 years less dialysis vintage compared to the HRG. The HRG had a higher percentage of subjects with diabetes (29.4% versus 20.8%), coronary artery disease (29.4% versus 18.9%) and vascular disease (31.4% versus 22.6%) compared to the LRG. Statistical significance for baseline imbalance was not performed because of the small sample size.

**Table 1:** Baseline demographic features and laboratory measurements by treatment allocation

	HRG	LRG
Number randomised (N)	51	53
Age (years) (Median, IQR)	60 (48,70)	65 (54,71)
Gender		
Male: Female	35:16	33:20
% of females:	31.4%	37.8%
Diabetes: No Diabetes:	15:36	11:42
Percentage with Diabetes:	29.4%	20.8%
Previous CAD:	15	10
No previous CAD:	36	43
Percentage with CAD:	29.4%	18.9%
Previous Vascular disease (including CAD):	16	12
No Previous vascular disease:	35	41
Percentage with previous vascular disease:	31.4%	22.6%
Duration on RRT: (Median, IQR)	2.5 years (1.5,5.0)	2.0 years (1.0,5.7)
Serum Phosphate (Mean, SD) (*P* = 0.1)	2.09 +/− 0.4 mmol/L (6.5 +/− 1.2 mg/dL)	2.2 +/− 0.4 mmol/L (6.8 +/− 1.2 mg/dL)
Corrected calcium (Mean, SD) (*P* = 0.4)	2.37 +/− 0.4 mmol/L 9.5 +/− 1.2 mg/dL	2.32 +/− 0.2 mmol/L 9.3 +/− 0.8 mg/dL
Serum PTH (pg/ml) (Mean, SD) (*P* = 0.8)	418 (273, 571)	392 (174, 675)
Serum Albumin (g/L) (Mean, SD) (*P* = 0.2)	32.7 +/− 7.7	34.6 +/− 6.9
Serum Cholesterol (mmol/L) (Mean, SD) (*P* = 0.9)	4.03 +/− 1.2	4.08 +/− 1.6

## Primary end-point

Primary end-point is the percentage of study participants achieving, and being maintained within, the higher and lower target ranges for phosphate, over the maintenance phase of the study [21]. Forty-seven subjects completed the titration phase in the HRG and 50 subjects in the LRG. At the end of titration, 29 HRG (61%) and 19 LRG subjects (38%) had achieved the target serum phosphate concentration, resulting in a mean phosphate of 2.00 ± 0.4 mmol/L (6.2 +/− 1.2 mg/dL) (HRG) and 1.63 ± 0.4 mmol/L (5.0 +/− 1.2 mg/dL) (LRG) (*p* < 0.01).

**Fig. 1:**
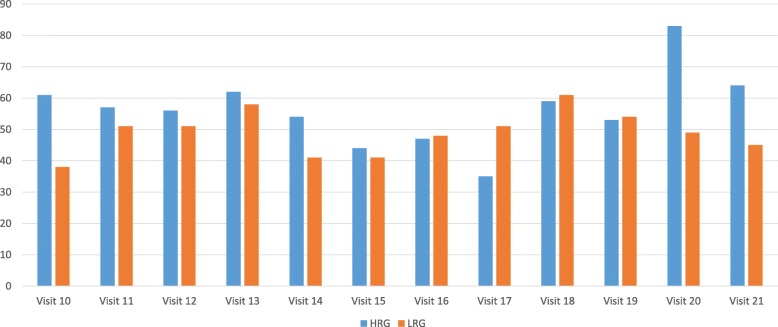
Percentage of subjects achieving target serum phosphate concentrations at sequential time-periods after randomization

Figure 1 shows the percentage of subjects achieving target serum phosphate concentrations at sequential time-periods after randomization.

**Fig. 2:**
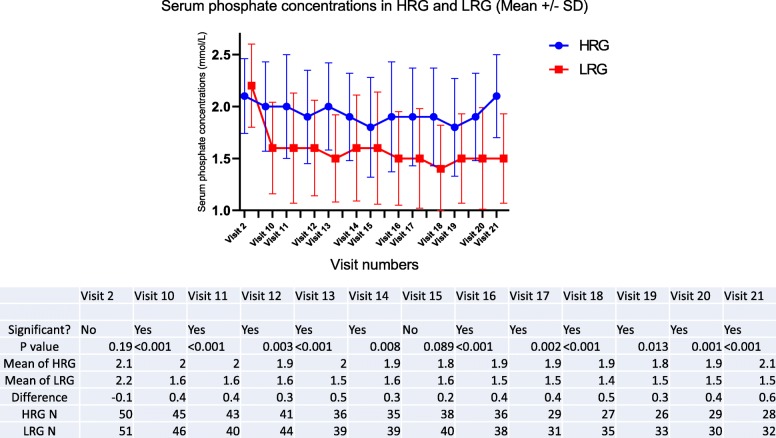
Significant separation in the serum phosphate concentrations between HRG and LRG (mmol/L)

At the start of washout, mean serum phosphate concentrations in the two groups was similar – 2.00 +/− 0.42 mmol/L (6.2 +/− 1.3 mg/dL) in the HRG versus 1.93 +/− 0.32 mmol/L (6.0 +/− 1.0 mg/dL) in the LRG. Following the washout period and prior to randomization mean serum phosphate concentrations in the two groups were not different (*p* = 0.15) – 2.1 +/− 0.36 mmol/L (6.5 +/− 1.1 mg/dL) in HRG Vs 2.2 mmol/L +/− 0.4 mmol/L (6.8 mg/dL +/− 1.2 mg/dL) in the LRG, but separated with a statistically significant difference at the end of titration, and maintained a separation of approximately 0.34 mmol/L (1.1 mg/dL) throughout the maintenance phase (Fig. 2), despite a large range.

## Secondary end-points

**Fig. 3:**
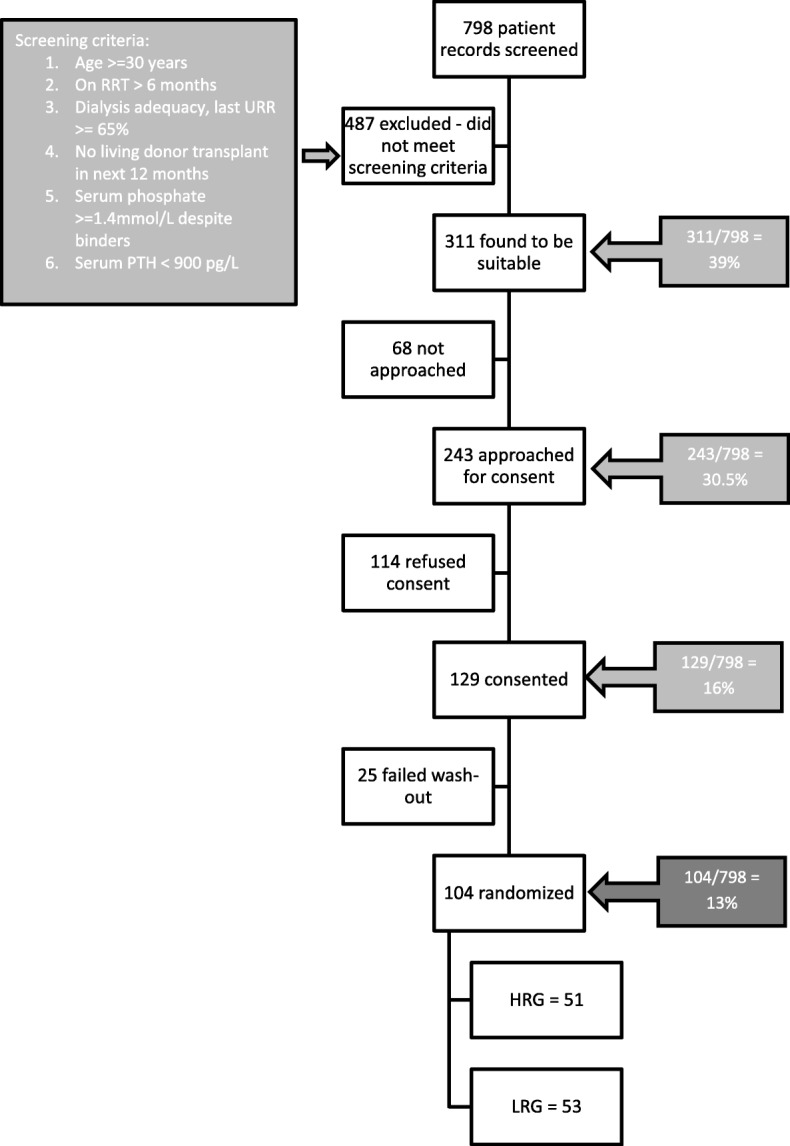
Flowchart of patient screening and recruitment

Seven hundred ninety-eight subject records were screened and 555 subjects excluded because they did not meet the screening criteria (Fig. 3). Fifty-three percent of the subjects approached gave consent (129/243). There was a difference in recruitment between the centers. Fifteen percent of the screened population was randomized at center 1 (76/494) versus 9% at center 2 (28/304). One hundred four subjects were randomized resulting in a recruitment rate of 13% of the dialysis population.

### Serious adverse events (SAEs)

Seventy-two SAEs were reported. Elective hospital admissions, planned out-subject procedures, elective dialysis catheter exchanges, blood transfusions for renal anaemia, hospitalisation for kidney transplantation and dialysis related headaches were not considered SAEs. After review by the investigators, 63 SAEs were determined to be in accordance with the trial criteria (Table 2).

**Table 2:** Number of significant adverse events in both groups with description of the events

Significant Adverse Events (SAEs)	Description of SAEs	HRG	LRG
Cardiovascular events	NSTEMI	2	0
	CVA	1	1
	Abdominal aortic aneurysm rupture	0	1
	Total number of cardiovascular events	3	2
Thrombotic	Fistula thrombosis	5	3
	DVT and PE	0	1
	Total number of thrombotic events	5	4
Sepsis		17	10
Metabolic bone complication		1	1
Miscellaneous	Blood transfusion	1	0
	Gastric ulcer	1	0
	Skin lesions	1	0
	Tumour	1	1
	Transplant kidney pain	0	1
	Sickle cell crisis	0	1
	RTA with spinal injury	0	1
	Non- cardiac	0	1
	Total miscellaneous	4	5
Mortality	Sepsis	4	0
	Cardiovascular event	3	1
	Sudden cardiac death	2	1
	Total mortality	9	2
Total SAEs		39	24

Nine deaths occurred in the HRG (17.6%) and 2 in the LRG (3.8%) - total mortality 11 (10.6%) over the 12 months. SPIRiT was not powered to examine differences in outcomes between the two groups. However, exploratory survival analysis was performed in an attempt to control for covariates known to be associated with mortality, because of the 4-fold higher mortality in the HRG. Time to mortality and first SAE (mortality OR cardiovascular event OR thrombo-embolic event) were considered for this analysis. Due to the small number of subjects in the study, only the hazard ratio and 95% confidence interval are reported. The unadjusted hazard ratio (HR) for mortality in the LRG, compared to the HRG, was 0.2 (95% CI 0.05, 0.98). Cox regression analysis adjusting for age, duration of dialysis, diabetes and pre-existent vascular disease yielded an HR of 0.19 (95% CI 0.04, 0.88) (Table 3).

**Table 3:** Adjusted and unadjusted hazard ratios

Outcome	Model	Hazard Ratio for LRG (vs. HRG)	95% Confidence Interval
Death	Unadjusted	0.21	0.05, 0.98
	Adjusted^a^	0.19	0.04, 0.88
Death + Cardiovascular Event	Unadjusted	0.34	0.13, 0.87
	Adjusted^a^	0.33	0.13, 0.86

^a^Adjusted for: Age, Duration of Dialysis, Diabetes and Previous CAD/ Vascular Disease

### Trial drop-out

The trial drop-out rate was 27% per annum with 65 subjects completing the full study - 35 LRG (66%) and 30 HRG (58.8%) (Table 4). More subjects withdrew consent in the LRG than in the HRG (9 versus 4). None of the subjects specified a reason for withdrawal of consent.

**Table 4:** Premature trial exits in both groups

Cause for trial-exit	Cause break-down	HRG	LRG
Number randomized		51	53
Successful transplant		4	4
Transferred to home dialysis		0	2
Withdrawn by study investigators	Hospital admission till study-end	2	0
	Intolerance to study medication	0	1
	High PTH at randomization	1	0
	Other reasons	1	0
	Total	4	1
Consent withdrawn by participant	Reason not given	0	6
	‘too much going on’, per patient	4	3
	Total	4	9
Total pre-mature trial exits		12	16
Mean duration in the study in months, SD		5.5 (3.9)	4.9 (3.4)

### Pill burden

**Fig. 4:**
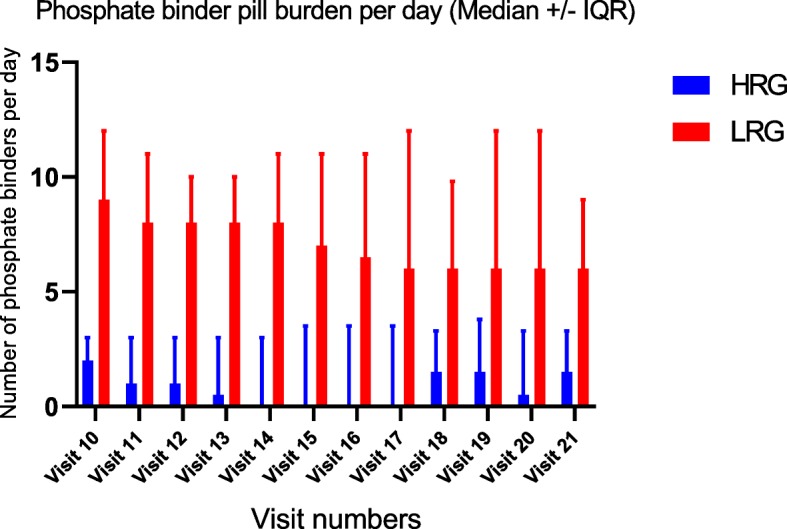
LRG had a substantially higher phosphate binder pill burden per day compared to the HRG

Subjects in the LRG took a median of 8 pills/sachets (IQR = 3 to 10) a day of phosphate binders (Fig. 4). The maximum number of binders per day that any LRG subject took was 17 pills/sachets per day. Only one patient in the HRG was on 9 sevelamer pills per day for the duration of the study, which was the maximum number of binders per day for any HRG subject (Median = 1, IQR 0 to 3).

### Alfacalcidol

**Fig. 5:**
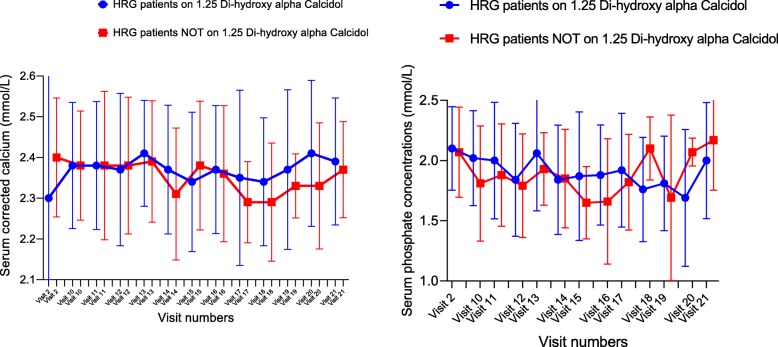
Serum concentrations of corrected calcium and phosphate were not significantly different in HRG patients on or off alfacalcidol

In the HRG, at Randomization (Visit 2), 33 patients were on alfacalcidol and 18 patients were not. No patients in this group stopped alfacalcidol during the duration of the study. There was no statistically significant difference in the mean serum phosphate levels or in the serum concentration of corrected calcium between these two groups at any visit (Fig. 5).

Table 5 shows the PTH concentrations in the HRG, in patients on alfa-calcidol and those who were not on alfa-calcidol. There was a significant difference in the median PTH between these patients (*P* = 0.07) at randomization but the difference disappeared as the study progressed.

**Table 5:** Serum PTH Concentrations in HRG patients on Alfaclacidol Versus HRG patients not on alfacalcidol

HRG	Visit 2	Visit 10	Visit 13	Visit 16	Visit 19
Yes - Alfacalcidol					
25% Percentile	341	276	271.7	175.4	214.1
Median	512.5	416	429.7	474.3	512
75% Percentile	827.5	676	693.2	752.6	842.1
No - Alfacalcidol					
25% Percentile	67	77	39	90	108
Median	290	229	237	262	250
75% Percentile	435	426	433	515	486
*P*-value	0.007	0.05	0.07	0.16	0.1

In the LRG, 43 patients were on alfa-calcidol at randomization and 8 were not. Alfa-calcidol was stopped for one patient during the study because of hypercalcemia. There was no statistically significant difference in the mean serum phosphate levels or in the serum concentration of corrected calcium between the LRG patients on or off 1, 25 alfa calcidol at any visit.

Table 6 shows the PTH concentrations in the LRG, in patients on alfa-calcidol and those who were not on alfa-calcidol. There was a significant difference in the median PTH between these patients (*P* = 0.002) at randomization but the difference could not be ascertained as the study progressed because there were only 8 patients not on alfa-calcidol.

**Table 6:** Serum PTH concentrations in patients on alfa-calcidol and in those not on alfa-calcidol in LRG

	Visit 2	10	13	16	19
Yes - Alfacalcidol					
25% Percentile	217	213.5	240.5	164.7	231.4
Median	497	410	398	407.1	368
75% Percentile	703	588.5	652.3	642.9	577
No - Alfacalcidol					
25% Percentile	30	189	120.5	88.1	87.62
Median	109	260	239.5	269.3	254.3
75% Percentile	302	277	299.1	427.2	482.9
*P*-value	0.002	0.29	0.1	0.3	0.4

The median weekly dosage of alfa-calcidol as 3mcg in both groups (IQR 1.6 to 3.5mcg in HRG and 1.8 to 3.5mcg in LRG) at randomization. There was no significant difference in the dosage between the two groups during the duration of the study.

## PTH and Cinacalcet

Table 7 shows serum concentration of PTH in HRG and LRG for the duration of the study. There is no significant difference in the median PTH between the two groups at any point during the study. However, one patient was taking oral Cinacalcet 30 mg a day at the start of the study – the patient continued with this dose for the study duration. Another patient in the HRG was started on Cinacalcit 30 mg during the maintenance phase – this patient underwent a deceased donor kidney transplantation and exited the study after a month of starting the Cinacalcit.

**Table 7:** Serum PTH concentrations in HRG and LRG during the study

	Visit 2	Visit 10	Visit 13	Visit 16	Visit 19
HRG					
25% IQR	176	229	13	0	151
Median	359	406	202	166	440
75% IQR	561	840	481	515	677
Number of values	46	39	31	29	18
LRG					
25% IQR	121	189	138	102	257
Median	351	359	314	336	384
75% IQR	632	534	467	532	575
Number of values	47	36	36	32	24
*P*-value	0.7	0.3	0.1	0.2	0.9

## Compliance with study targets

Four hundred thirteen blood samples were collected in the HRG during the maintenance period, i.e., visit 10 onwards. Individual patients’ serum phosphate concentration varied quite widely, but considering all blood results collectively, 55% were within the treatment target range of 1.8 to 2.4 mmol/L (5.6 to 7.4 mg/dL). Serum phosphate concentration was below the target in 134/413 samples and above the target in 55/413 blood samples. Only one patient remained within in the target range for the entire duration of the study.

Four hundred forty-nine blood samples were collected in the LRG during the maintenance period, i.e., visit 10 onwards. Forty-five percent of all serum phosphate results were within the treatment target range of 0.8 to 1.4 mmol/L (2.4 to 4.3 mg/dL). Serum phosphate concentration was below the target in 8/449 samples and above the target in 246/449 blood samples. Only two patients remained within the target range for the entire duration of the study.

## Other biochemical parameters

**Fig. 6:**
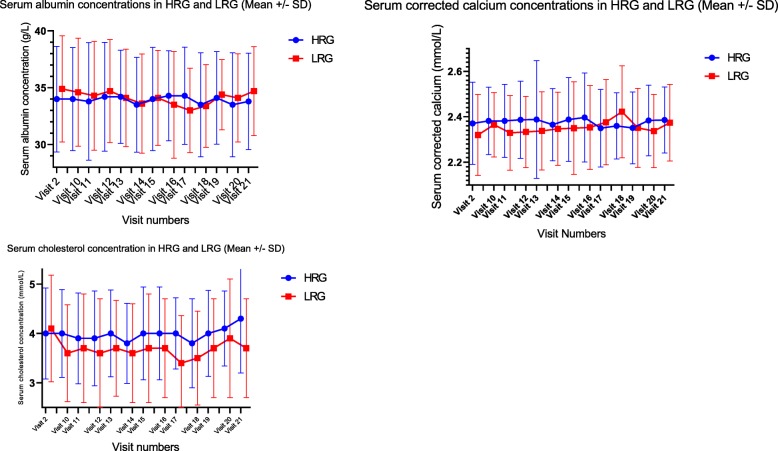
Serum albumin, corrected calcium and cholesterol concentrations in the HRG and the LRG. Visit 2: Randomization visit, start of Titration to treatment targets for serum phosphate concentration Visit 10: 8 weeks after visit 2 – End of Titration. Visit 11 to Visit 21 are visits during the maintenance phase, each visit 4 weeks apart

Despite obtaining a clear separation in the serum concentration of phosphate levels between the two groups, mean serum calcium concentrations remained steady with no significant differences between the two groups (Fig. 6) – it remained steady at 2.3 +/− 0.2 mmol/L in both the groups. Serum albumin showed no significant difference between the two groups and the mean remained at 35 +/− 4 g/L through the maintenance period. Serum Cholesterol dropped significantly ion the LRG from 4.1 +/− 0.9 mmol/L at randomization to 3.6 +/− 0.9 mmol/L at the end of titration. At the end of titration, mean cholesterol concentrations in the HRG had remained the same as that at randomization, 4.0 +/− 1 mmol/L. Hence, at visit 10, there was a statistically significant lowering of cholesterol in the LRG with *p* < 0.04. But the statistical significance did not persist beyond visit 10.

## Discussion

This study demonstrates that it is possible to recruit and randomize dialysis patients to two different serum phosphate concentrations, and maintain them over 10 months, despite the HRG being above the range recommended by current guidelines. Approximately 50% of the SPIRiT subjects in either group achieved the trial target range of serum phosphate at multiple-time points in the trial and a difference of 1.1 mg/dL (0.34 mmol/L) between the HRG and the LRG was achieved and maintained during the 10-month follow-up. Having two mutually exclusive *target ranges* rather than *target concentrations* ensured separation of the groups’ mean phosphate values. Block and colleagues [3] studied retrospective data for 40,538 in-center hemodialysis subjects sampled from the Fresenius medical care North America patient statistical profile system, and stratified serum phosphate concentrations into 8 categories of 1 mg/dL increments. With multivariable adjustment, the relative risk of death increased from 1.0 for serum phosphate concentration of 4.0 to 5.0 mg/dL to 1.1, > 1.2, 1.4, 1.7 and 2.0 at serum phosphate concentrations of 5–6, 6–7, 7–8, 8–9 and > 9 mg/dL respectively. The phosphate separation achieved in the SPIRiT study was 0.34 mmol/L - equivalent to 1.05 mg/dL, and based on such retrospective data, would be adequate to show a difference in clinical outcomes, if any, in a large-scale RCT.

We did not achieve our goal of serum phosphate between 0.8 to 1.4 mmol/L (2.5–4.3 mg/dL) as less than 1/2 subjects in that group achieved that goal and the mean level was 1.5 +/− 0.4 mmol/L (4.6 +/− 1.2 mg/dL). We did achieve a difference between the 2 groups as the high phosphate group had a mean of 2.0 +/− 0.5 mmol/L (6.2 +/− 1.5 mg/dL) and still did not have a high percentage in the range. There was only one other study which attempted to randomize a similar number of patients to two different serum phosphate ranges [23]. This study was started after we started to recruit for the SPIRiT study, and had a much shorted follow-up period of 26 weeks. The investigators further used calcium carbonate, a calcium containing phosphate binder. While it can be argued that there is no RCT evidence showing increased mortality in dialysis patients with calcium containing binders, there is evidence that they increase the risk of vascular calcification [24]. This is a compelling reason to not use these in the larger study. Further, there was no washout period which makes it possible that patients were included in the study who did not need to be on the phosphate binders in the first place.

Whilst it may be possible to achieve specific concentrations of phosphate over a specified time period, a larger study will depend on the willingness of physicians and patients to participate. Consequently, the secondary end points of this study examine this. Of the 22 local nephrologists, only 2 raised concerns regarding their patients taking part in the study, and this was not a clinical concern but related to the effect higher phosphate concentrations might have on their unit’s adherence to national targets. They were anxious that a deterioration in reported results might attract criticism – an understandable concern in the current target-driven culture. They agreed to take part on condition that that year’s UK Renal Registry report carried a comment about the possible effects of the SPIRiT study on compliance with targets.

Willingness of patients to participate, once approached, was high with 53% (129/243) of the eligible participants consenting. Local research practices and trial personnel appear to have had a significant effect on recruitment with 16% of screened subjects at center 1 consenting versus 9% at center 2. This was most likely due to the study clinician being primarily based at center 1. In a much larger study it is unlikely that the majority of centers would have a full time research clinician available and therefore the lower recruitment rate may be more representative of ‘real life’. In addition, the pace of recruitment at the two centers varied (8.4 subjects per week recruited versus 4.3 per week respectively) probably for similar reasons.

At randomization, 29.4% of the HRG had diabetes versus 20.8% of the LRG. More HRG had previous coronary artery disease (29.4% of HRG versus 18.9% of LRG) and more vascular disease (31.4% HRG versus 22.6% LRG). We did not stratify for the cardiovascular risk factors at randomization because of the small sample size. In a larger study, it is likely that an adequate sample size will resolve these discrepancies. However, as the EVOLVE [25] study demonstrated, a large sample size may not always guard against bias, and the larger study would need stratified randomization for established cardiovascular risk factors in dialysis patients.

The overall trial drop-out rate of 27% per annum was comparable to other interventional RCTs in dialysis patients. The HEMO study randomized 1846 patients to high flux or low flux dialysis and followed them up for a mean of 2.84 years. Five hundred ninety patients dropped out over the course of the study which gives a drop-out of 33% during the study [26]. On the other hand, the EVOLVE study randomized 3883 dialysis patients with secondary hyperparathyroidism to cinacalcet group or placebo with to examine differences in clinical outcomes. Seventy-eight percent of the subjects in the cinacalcet group and 61% in the control group stopped the study medication for non-protocol-specific reasons resulting in a crossover between the groups [27]. In the SPIRiT study, the drop-out rate in the LRG and HRG was 7/51 (13%) and 10/53 (19%) respectively. Consent withdrawal was higher in the LRG (4 vs 9). Although subjects did not give clear explanations for their withdrawal it seems likely that pill burden may have been a factor, with all LRG consent withdrawals happening in the first 4 months of the trial. Interestingly during the washout period at the start of the study 2 patients said they felt so much better having stopped their binders that they refused to restart and were withdrawn from the study.

Only non-calcium containing phosphate binders were used in this study, and at the start of the trial in 2013, availability was limited to lanthanum and sevelamer [13]. Any subject requiring high dose phosphate binders and intolerant of lanthanum was limited to sevelamer tablets which could result in a pill-burden of up to 15 tablets a day. Since the completion of this study, other non-calcium binders have become available which might make a larger study easier by providing subjects with more choice [28] and lower pill burden.

One patient in the HRG was on Cinacalcet 30 mg a day at the start of the trial and stayed on it for the duration of the study. One other patient needed to be started on Cinacalcet during the study. Hypercalcemia was not a particular problem since only non-calcium containing phosphate binders were used. The dose of 1.25 alfa–calcidol was determined by the clinical care team and the presence or absence of alfa-calcidol therapy did not make an impact on the calcium and phosphate concentrations in the trial. However, the availability of alfa calcidol ensured that hypocalcaemia was adequately treated. With the availability of non-calcium containing phosphate binders, it is possible to deliver effective phosphate binding without the need for calcium loading; It is also possible to control PTH with calcimimetics despite a higher serum phosphate concentration – the availability of medication to address these mineral disorders in isolation makes it possible to design and conduct such studies.

Although mortality in the HRG (9/51) was substantially higher than in the LRG (2/53), the overall mortality for the study was 10.6%, which compares favourably with the UK Renal Registry annual mortality rate of 11% [29]. A similar rate would be expected in a small trial such as this, with standard exclusion criteria possibly creating a selection bias towards subjects with fewer comorbidities. However, as the study progressed the difference in death rate between LRG and HRG increased noticeably, causing the Safety Monitoring Committee to scrutinise the data in ever greater detail each month. The Committee satisfied itself that the difference in mortality was likely to have occurred by chance plus the notable imbalance in risk factors at randomisation. Prevalence of diabetes, pre-existing coronary artery disease and vascular disease were significantly higher in the HRG. The trial was not powered to examine clinical outcomes such as mortality and cardiovascular events.

LRG and HRG achieved the treatment target ranges just about 50% of the time. Only 3 patients stayed in their respective target ranges for the entire duration of the study. A high pill burden which can enhance poor adherence is very likely the cause for the LRG patients not always achieving their target ranges. It is unclear why the serum phosphate concentrations were below the target range in the HRG over 30% of the time (134/413 samples in the maintenance period). This happened despite careful screening and washout ensuring that no patient who did not need a phosphate binder at baseline, was inadvertently recruited. One possibility is that the years of educating the dialysis staff and the patients about the benefit of low phosphate inadvertently introduced a bias where in the HRG patients practiced extra care about maintaining a low phosphate diet during the study period, since they were not on phosphate binders. In the design of the larger study, it is important to include a regular diet diary monitoring to see if patient and provider biases play a role in tighter phosphate control in the HRG.

Oral phosphate binder tablets can have significant gastro-intestinal side effects such as bloating which can lead to reduced oral intake and poor nutrition. We monitored serum albumin as a surrogate marker of nutrition, and did not find any significant reduction in the LRG which had the largest pill burden. As expected, the LRG had a lower serum cholesterol concentration (though not statistically significant). Sevelamer is a polymer which is known to cause a reduction in cholesterol levels.

Based on the results of this study it is possible to undertake a sample size calculation for a large scale, longer term, randomised controlled trial. The sample size calculation projects an annual incidence of 15% for the composite of non-fatal cardiovascular events and mortality in dialysis patients with 80% power to detect a 5% reduction in event rate. One thousand three hundred seventy-two patients will need to be randomized to give 80% power to detect a 5% reduction in event rate. To detect this difference, *all* patients would require to complete the trial. Allowing for trial attrition from reasons unrelated to the primary endpoint, the calculated sample size would need to be adjusted. For a 20% non-primary end-point attrition, the sample size increases by 20% resulting in 1716 (858 per group).

## Conclusion

This study demonstrates that it is possible to randomise dialysis patients to different concentrations of phosphate control – a hope first suggested in the introductory sections of guidelines published as long ago as 2003 (KDOQI) and 2009 (KDIGO). Though the TARGET study showed the possibility of randomization, SPIRiT study demonstrated that a clinically significant difference in serum phosphate concentrations can be maintained in the two groups over a prolonged period of time (1 year) which is necessary to study differences in clinical outcomes in the two groups. Both physicians and patients agreed to participate in numbers that suggest a larger scale study is possible. Despite this being an interventional RCT, 43% of the eligible target population were successfully consented, and the drop-out rate was comparable to published large scale, long-term dialysis studies. A clinically significant separation of mean serum phosphates was achieved between the groups representing a possible increased relative risk of 0.2 based on published observational data.

Whilst the number of deaths in the HRG was noticeably higher than in the LRG the study is under-powered to evaluate this outcome.

These results suggest that a similar but larger 2 year study is indeed possible. Based on a lower conversion rate of 10% from screening to randomisation, screening of 17,160 patients would be expected to result in 1716 consents; 858 patients in each group. One thousand two hundred fifty-three patients would remain after 12 months and approximately 915 in follow-up after 24 months. Such a study would counter “the lack of patient-centered outcomes as end points in the majority of trials in this field” as highlighted by KDIGO.
